# Effects of *Xiaoshuan* enteric-coated capsule on neurovascular functions assessed by quantitative multiparametric MRI in a rat model of permanent cerebral ischemia

**DOI:** 10.1186/s12906-016-1184-z

**Published:** 2016-07-08

**Authors:** Jian Zhang, Haiyan Zou, Qiuxia Zhang, Lei Wang, Jianfeng Lei, Yali Wang, Junyao Ouyang, Yi Zhang, Hui Zhao

**Affiliations:** School of Traditional Chinese Medicine, Capital Medical University, Beijing, 100069 People’s Republic of China; Beijing Key Lab of TCM Collateral Disease Theory Research, Beijing, 100069 People’s Republic of China; School of Chinese Medicine, Hongkong Baptist University, Hongkong, People’s Republic of China

**Keywords:** Ischemic stroke, Middle cerebral artery occlusion, Magnetic resonance image, Neurovascular unit, Cerebral blood flow, *Xiaoshuan* enteric-coated capsule

## Abstract

**Background:**

*Buyang Huanwu* Decoction (BYHWD) is a Traditional Chinese Medicine (TCM) formula for treating stroke-induced disability. *Xiaoshuan* enteric-coated capsule (XSECC), derived from the formula BYHWD, is a drug approved by the China Food and Drug Administration (CFDA) for stroke management. To further investigate the potential protective effects of XSECC on neurovascular functions, we endeavour to monitor the neurovascular functions using multimodal magnetic resonance imaging (MRI) and evaluated histopathological changes of neurovascular unit (NVU) after stroke.

**Methods:**

Ischemic stroke was induced by permanent middle cerebral artery occlusion (pMCAO). XSECC (420 mg/kg) was orally administered 2 h after stroke and daily thereafter. T2-weighted imaging (T2WI), T2 relaxometry mapping and diffusion tensor imaging (DTI) were used to measure cerebral infarct volume, edema and white matter fiber integrity, respectively. Neurochemical metabolite levels were monitored by ^1^H-magnetic resonance spectroscopy (^1^H-MRS). Arterial spin labeling (ASL) – cerebral blood flow (CBF) measurements and structural magnetic resonance angiography (MRA) images provided real-time and dynamic information about vascular hemodynamic dysfunction on the 3rd, 7th and 14th days after pMCAO. At the last imaging time point, immunohistochemistry, immunofluorescence as well as transmission electron microscopy (TEM) were used to test the microscopic and ultrastructural changes of NVU.

**Results:**

T2WI, T2 relaxometry mapping and Fractional anisotropy (FA) in DTI showed that XSECC significantly reduced cerebral infarct volume, relieved edema and alleviated nerve fiber injuries, respectively. ^1^H-MRS provided information about improvement of neuronal/glial metabolism after XSECC treatment. Moreover, ASL – CBF measurements combined with MRA showed that XSECC significantly increased CBF and vascular signal strength and alleviated ischemia-induced morphological changes of arteries in ischemic hemisphere within 14 days after stroke. In addition, neuron specific nuclear protein (NeuN), glial fibrillary acidic protein (GFAP), CD34 staining and TEM detection indicated that XSECC not only ameliorated neuronal injury, but also reduced endothelial damage and inhibited astrocyte proliferation.

**Conclusions:**

Our results suggested that XSECC has multi-target neurovascular protective effects on ischemic stroke, which may be closely correlated with the improvement of cerebral blood supply and neuronal/glial metabolism.

## Background

Stroke is the third most common cause of death in the world, and one of the leading causes of long-term disability. About 80 % of strokes are caused by ischemia resulting from an obstruction of blood flow [1, 2]. Many neuroprotectants showed promises in animal models, but were not effective in clinical practice. Effective treatment options for stroke patients are limited [3]. Neurovascular unit (NVU) is a functionally and structurally interdependent multicellular complex which consists of neurons, endothelial cells, astrocytes and basal lamina, act as an intricate network to maintain a homeostatic neuronal microenvironment [4, 5]. The complex contact of NVU was badly damaged after ischemic stroke, which leads to neurological dysfunction [6, 7]. However, many conventional therapeutic agents only focus on maintaining neuronal function but neglecting other components of the NVU [8]. Therefore, a new neurovascular protective therapy capable in restoring tissue perfusion, preserving vascular integrity, and minimizing neuronal cell death is urgently needed for ameliorating ischemic insult [9, 10].

*Buyang Huanwu* Decoction (BYHWD), a well-known traditional Chinese herbal formula, has been used for the treatment of paralysis and stroke since the Ming Dynasty [11]. Studies showed that BYHWD exhibited neuroprotective effects by activating pro-survival signaling cascades in neurons, inhibiting inflammatory cascade reactions and suppressing apoptosis signals [12–18]. Studies also showed that BYHWD exhibited potential vascular-protective effects by improving hemorheological disorders, inhibiting arterial thrombosis, and suppressing intimal hyperplasia [19, 20]. The multi-component and multi-target characteristics of BYHWD make it an attractive candidate for neurovascular protective agent in the treatment of stroke. However, the development of Traditional Chinese Medicine (TCM) formulae has been hampered by their inconsistent preparation, unstable quality, and lack of uniform standards. Recently, new medicinal preparation methods are being used to tackle these problems. *Xiaoshuan* enteric-coated capsule (XSECC), is a novel preparation of the traditional formula BYHWD, has received the permission of the Chinese State Food and Drug Administration (CFDA) to access the free market (drug approval number Z20000025). Although recent studies with permanent middle cerebral artery occlusion (pMCAO) animal models suggested therapeutic benefit of XSECC [21, 22], the effects of XSECC on neurovascular functions during ischemia remain to be evaluated.

It is well accepted that the evolution of ischemic brain damage is a heterogeneous and dynamic process [23]. In experimental stroke research, new potential neuroprotective treatments on brain damage are commonly evaluated by using 2, 3, 5-Triphenyltetrazolium chloride staining, histopathological examination and electron microscopic observation. However, these measurements can only be applied at a single time point and are insufficient for dynamically assessing the progressive changes of neurovascular functions during the entire phase of ischemia [24]. It is highly desirable to utilize a method that could continuously monitor hemodynamic and neural responses in vivo with high spatial resolution. Magnetic Resonance Imaging (MRI) is a powerful and noninvasive technique that provides large scale measurements of neural functions compared to the current detection techniques [25]. Using multiple MRI modalities is important to understand the alterations in neurovascular status and suitable for the evaluation of NVU protective and therapeutic approaches [26]. In the present study, we implemented a multimodal MRI design, including T2-weighted imaging (T2WI), diffusion tensor imaging (DTI), ^1^H-magnetic resonance spectroscopy (^1^H-MRS), magnetic resonance angiography (MRA) and arterial spin label (ASL) perfusion MR imaging, to longitudinally monitor and track neurovascular functions at different - time points and evaluate the potential protective effects of XSECC on NVU, followed by histopathological examination at the last time point.

## Methods

### Reagents

Hydroxysafflor yellow A (batch no. MUST-13121913), paeoniflorin (batch no. MUST-13113009), calycosin-7-O-β-D-glucoside (batch no. MUST-14031314), calycosin (batch no. MUST-13081501), and formononetin (batch no. MUST-14030710) were purchased from Chengdu Must Bio-technology Co. Ltd. (Sichuan, China). Ononin (batch no. 130507) was obtained from Chengdu Pufei De Biotech Co. Ltd. (Sichuan, China). The purity of all the above chemical reference substances were determined higher than 98 %. Rabbit anti-rat neuron specific nuclear protein (NeuN) and mouse anti-rat glial fibrillary acidic protein (GFAP) were bought from Millipore Co. Ltd (USA). Rabbit anti-rat CD34 was purchased from Abcam (UK).

### Preparation of XSECC

XSECC (batch no. 20130407) was supplied by Sanmenxia Sinoway Pharmaceutical Co. Ltd. (Henan, China). The formula comprises 7 herbs as shown in Table 1. Herbal materials were authenticated by the Sanmenxia Sinoway Pharmaceutical Co. Ltd. and the herbal samples with voucher number 2013002 were deposited in the Beijing Key Lab of TCM Collateral Disease Theory Research, Capital Medical University, China. The preparation of XSECC is described as follows: air-dried materials (6.30 kg) were refluxed twice with 50 % ethanol (1:10 for the first extraction and 1:8 for the second extraction, w/v) for 2 h and filtered. The filtrates were concentrated and evaporated under vacuum to yield the extract. Then, the extract was ground into fine powder and filled into capsules. One gram of the capsule powder was produced from 6.3 g crude herbs.

**Table 1:** Pharmaceutical ingredients of Xiaoshuan enteric-coated capsule

Latin name	Species	Family	Part used
Radix Astragali	*Astragalus membranaceus* (Fisch.) Bge.var.*Mongholicus* (Bge.) Hsiao	*Leguminosae*	Roots
Pheretima	*Pheretima aspergillum* (E. Perrier)	*Megascolecidae*	Bodies
Radix Paeoniae Rubra	*Paeonia lactiflora* Pall.	*Paeoniaceae*	Rhizomes
Radix Angelica Sinensis	*Angelica sinensis* (Oliv.) Diels	*Umbelliferae*	Roots
Rhizoma Ligustici Chuanxiong	*Ligusticum chuanxiong* Hort.	*Umbelliferae*	Rhizomes
Semen Persicae	*Prunus persica* (L.) Batch	*Rosaceae*	Seeds
Flos Carthami	*Carthamus tinctorius* L.	*Compositae*	Flowers

Note: The ratio of these herbs was 71:4:7:7:4:4:4

### Quantitative analysis of XSECC by HPLC

The contents of six main components in XSECC, including hydroxysafflor yellow A, paeoniflorin, calycosin-7-O-β-D-glucoside, ononin, calycosin and formononetin, were determined using an Agilent 1260 liquid chromatography system (Agilent Technologies, USA), consists of an autosampler, a quaternary pump, a diode-array detector, and a column temperature controller. Agilent Chem Station software version B.04.03C was used for data collection and analysis. Before the chromatographic analysis, 0.4 g capsule powder was ultra-sonicated (300 W, 40 kHz) with 50 % methanol (4 mL) at 30 °C for 30 min, then dilute with 50 % methanol to 5 mL and filtrated through 0.45 μm membrane filter. The analysis was performed on a Zorbax SB-C18 (250 mm × 4.6 mm, 5 μm, Agilent) column at 30 °C, using a gradient mobile phase consisting of acetonitrile (solvent A) and 0.1 % phosphoric acid (v/v, solvent B). The linear gradient was as follows: 0–15 min, 99 - 90 % B; 15–45 min, 90 - 60 % B; 45–60 min, 60 - 30 % B; 60–80 min, 30 - 0 % B. The flow rate was 1.0 mL/min. The wavelength was set at 230 nm (for paeoniflorin), 254 nm (for calycosin-7-O-β-D-glucoside, formononetin, calycosin and ononin) and 403 nm (for hydroxysafflor yellow A). The injection volume was 5 μL. Under the current conditions, HPLC peaks for all the quanlity control components had very good separation. The contents of hydroxysafflor yellow A, paeoniflorin, calycosin-7-O-β-D-glucoside, formononetin, calycosin and ononin were 1.24 mg/g, 2.12 mg/g, 1.04 mg/g, 0.42 mg/g, 0.13 mg/g and 0.08 mg/g, respectively.

### Animals and stroke induction

Male Sprague–Dawley rats, aged 10 weeks (280 ± 20 g) were obtained from the Vital River Laboratory Animal Technology Co. Ltd. (Beijing, China). The room in which rats were housed was 21 ± 2 °C and 55 ± 5 % relative humidity with artificial 12:12 h equivalent light–dark cycles. All experimental procedures were carried out according to the National Institute of Health Guide for the Care and Use of Laboratory Animals, and approved by the local Ethical Committee at the Capital Medical University (Permit Number: No. 2011-X-001). Attempts were made to minimize suffering and reduce the numbers of animals used wherever possible.

The pMACO model was established as previously described [27]. The right common carotid artery, external carotid artery (ECA) and internal carotid artery (ICA) were exposed. The ECA and ICA were temporarily clamped using microsurgical clips. A 4–0 monofilament nylon suture (Beijing Sunbio Biotech Co. Ltd., China) was inserted into the ICA and gently advanced from the lumen of the ICA until it blocked the origin of the MCA. Sham-operated rats were manipulated in the same way, without the MCA occlusion.

### Drug administration and animal grouping

Twenty-six rats were subjected to the same MCA occlusion procedure and were randomized to the Stroke (*n* = 16) or to the XSECC/Stroke group (*n* = 10). XSECC was dissolved in sterile saline. Our previous results indicated that a dose of 420 mg/kg/day exhibited maximal protective effects in the treatment of ischemic injury in rats [21]. Therefore, in this study, XSECC (420 mg/kg/day) was orally administered 2 h after stroke induction, and then once daily for 14 days. Rats in the Sham group and the Stroke group (*n* = 4) were administered with normal saline (10 ml/kg/day).

### MRI acquisition and data analysis

The rats were subjected to MRI examination on the 3rd (4 rats in the Sham group, 7 in the Stroke group and 7 in the XSECC/Stroke group), 7th (4 rats in the Sham group, 6 in the Stroke group and 6 in the XSECC/Stroke group) and 14th days (4 rats in the Sham group, 3 in the Stroke group and 5 in the XSECC/Stroke group) after pMCAO. MRI was conducted on a 7.0 T magnet (Bruker, Pharma Scan, Germany), equipped with an actively shielded gradient system (14 cm inner diameter). Rats were anesthetized using facemask inhalation of 1.8 % isoflurane by isoflurane anesthesia system (JD Medical Dist. Co. Inc., USA).

#### Evaluation of cerebral infarct volume

T2WI was used for measuring cerebral infarct volume [28]. T2WI images of coronal slices were acquired with a fast spin-echo pulse sequence, repetition time (TR) = 4400 ms, echo time (TE) = 45 ms, field-of-view (FOV) = 3 × 3 cm, matrix size = 256 × 256 and slice thickness = 0.7 mm. The hyperintense areas in each coronal slice were analysed by Image J software. The cerebral infarct volume was determined as the sum of the hyperintense areas in each slice multiplied by the slice thickness [28].

#### Determination of cerebral edema

T2 relaxometry mapping could provide T2 values which are used to evaluate post-ischemia cerebral edema [29, 30]. T2 relaxometry maps were acquired using multi-slice-multi-echo (MSME) sequences by applying 16 TEs, from 11 to 176 ms, TR = 2500 ms, FOV = 3.3 × 3.3 cm, and Matrix size = 256 × 256. In all animals, the T2 values were calculated for ipsilateral cortex and striatum based on T2 relaxometry maps that were reconstructed by paravision version 5.1 software (Bruker, Pharmascan, Germany).

#### Assessment of nerve fiber injuries

Fractional anisotropy (FA) is one of the most sensitive DTI parameter for detecting the integrity of white matter fibers after cerebral damage [31]. DTI was acquired using an axial single-shot spin echo-planar sequence with a TR/TE = 6300/25 ms, 30 diffusion encoding directions, two b values = 0 and 1000 s/mm^2^. FA images were reconstructed with paravision version 5.1 software (Bruker, Pharmascan, Germany). FA was measured in the following four regions: ipsilateral cortex and striatum, contralateral cortex and striatum. The relative FA (rFA) was calculated by comparing the parameters in the lesion ROIs (Regions of interest) with the mirrored ROIs of the contralateral side [32].

#### Measurement of cerebral perfusion

CBF images are ideal to evaluate the extent of cerebral perfusion after pMCAO. CBF images were obtained from continuous ASL with echo-planar imaging fluid-attenuated inversion recovery (EPI-FLAIR) sequences. CBF images were reconstructed with paravision version 5.1 software (Bruker, PharmaScan, Germany). Acquisition parameters were TR/TE = 18000/25 ms, FOV = 3.0 × 3.0 cm, matrix size = 128 × 128, and the number of excitations (NEX) = 1. CBF was measured in the ipsilateral and contralateral hemispheres. Then, the relative cerebral blood flow (rCBF) was calculated using the equation: rCBF = (ipsilateral CBF/contralateral CBF) × % [33].

#### Measurement of cerebral arteries dynamic changes

MRA images performed the morphological alterations of arteries. MRA images were acquired by time-of-flight (TOF) angiography with a three-dimensional (3D) Fast Low Angle Shot (FLASH) method. The scan parameters were set as follows: TR/TE = 15/2.5 ms, FOV = 3.5 × 3.5 × 4.0 cm, Matrix size = 256 × 256 × 128, and NEX = 1. MRA images were reconstructed with paravision version 5.1 software (Bruker, Pharmascan, Germany). The signal intensity of ipsilateral cerebral artery was analysed by Image J software [34].

#### Assessment of neuronal/glial metabolism

^1^H-MRS indicates the information of neuronal/glial metabolism [35, 36]. ^1^H-MRS of the rat brain was acquired using a PRESS sequence with the following parameters: TR/TE = 1500/135 ms, voxel = 5 cm × 4 cm × 4 cm. ^1^H-MRS image analysis was performed off-line using topspin 2.0 software (Bruker, Pharmascan, Germany). For each rat, N-acetyl aspartate (NAA) to creatine (Cr) ratio (NAA/Cr) and choline (Cho) to Cr ratio (Cho/Cr) were recorded. Since Cr methyl resonance at 3.02 ppm value is not affected by various pathologies, it was used as an internal reference to calculate the relative levels of other metabolites [37].

### Tissue processing

At the end of the MR examination, rats were perfused transcardially with saline solution, followed by 1 % glutaraldehyde and 4 % paraformaldehyde in 0.1 mol/L phosphate buffer. Subsequently, the brains were removed, dissected on ice, and 1.0 mm^3^ parietal cortex sections were prepared, which were located 1.7 – 0.7 mm rostral to the bregma, and then post-fixed in 2.5 % glutaraldehydeand processing for transmission electron microscopy (TEM). Sequential sections between the bregman 0.7 mm and 4.8 mm of each animal were fixed in 4 % phosphate-buffered paraformaldehyde for immunofluorescence and immunohistochemistry.

### Transmission electron microscopy

As reported previously [38], the alteration of the NVU ultrastructure after cerebral ischemia was detected by TEM. After fixation, the samples were dehydrated in graded acetone and embedded in Epon 812. The ultrathin sections were stained with 4 % uranyl acetate-lead citrate and examined by TEM (JEM-1230, Jeol, Japan).

### Immunofluorescence and immunohistochemistry

The expression levels of NeuN, GFAP and CD34 are correlated with the pathological changes of neurons, astrocytes, and capillary endothelial cells, respectively [39, 40]. NeuN and GFAP immunofluorescence staining were performed as reported elsewhere [39]. Coronal sections were incubated at 4 °C for 40 h with anti-GFAP (1:200) and anti-NeuN (1:100). Subsequently, sections were incubated for 2 h at 37 °C with fluorophore-conjugated secondary antibodies. Immunohistochemistry was performed as previously described for CD34 staining [40]. Brain sections were incubated with rabbit anti-CD34 (1:100) at 4 °C for 40 h. Then, sections were incubated with biotin-labeled secondary antibody (sheep anti-rabbit IgG) at 37 °C for 60 min. Color development was accomplished by exposure to 3,3’–diaminobenzidinetetrahydrochloridehydrate for 40 s. Negative control sections were used without labeling with the primary antibody.

Finally, sections were cover-slipped and visualized with a light microscope (40× objective) (Nikon Eclipse80i, Japan). We quantitatively analyzed labeled cells as previously described [41] with NIS-elements basic research software (Nikon, Japan). In all slices, four randomly fields of peri-infarct regions of the parietal cortex in each slice were quantified by a blinded observer. The expression levels of NeuN, GFAP and CD34 were expressed as integrated optical density (IOD) values.

### Statistics analysis

All statistical analyses were carried out by using SPSS 11.5 software (SPSS, Chicago, IL, USA). Data are expressed as means ± standard error of the mean (SEM). The one-way analysis of variance (ANOVA) followed by a Fisher’s LSD test was used for within-group comparisons. For two group comparisons, the independent sample Student’s *t*-test was performed to compare the differences between the Stroke group and the XSECC/Stroke group. A significant difference was considered if the *p*-value was less than 0.05.

## Results

### XSECC reduced the cerebral infarct volume

**Fig. 1:**
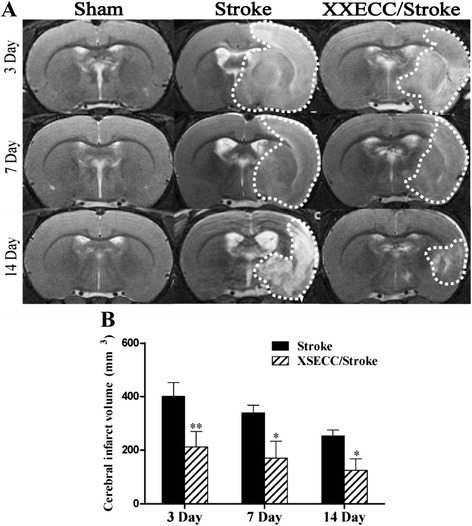
T2-weight images on the 3rd, 7th and 14th days after pMCAO. **a** Representative T2WI images of the Sham, the Stroke and the XSECC/Stroke group. The high signal intensity on T2-weighted images as indicated by the white dotted line. **b** Comparison of cerebral infarct volume between the Stroke group and the XSECC/Stroke group. Values are means ± SEM. *P < 0.05, **P < 0.01 vs the Stroke group

T2-weighted images showed abnormal hyperintense in the ischemic hemisphere involving the striatum and some adjacent cortical regions (parietal cortex and piriform cortex) after pMCAO (Fig. 1a). Furthermore, XSECC significantly decreased the cerebral infarct volume as compared to the Stroke group. These results demonstrated that XSECC is a useful drug to mitigate brain damage after pMCAO (Fig. 1b).

### XSECC decreased cerebral edema

**Fig. 2:**
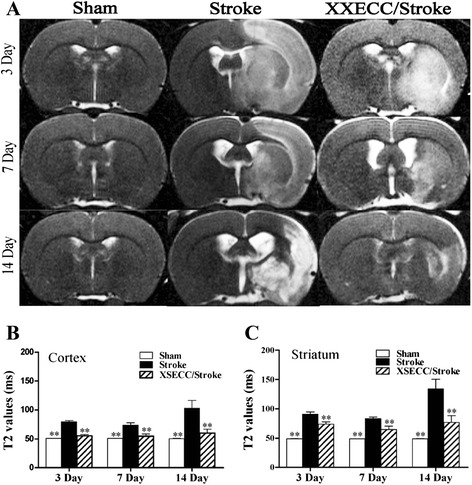
T2 mapping images on the 3rd, 7th and 14th days after pMCAO. **a** Representative T2 mapping images of the Sham, the Stroke and the XSECC/Stroke groups. T2 values in the (**b**) cortex and (**c**) striatum. Values are means ± SEM. **P < 0.01 vs the Stroke group

The abnormal high signal intensity was observed in ischemic hemisphere on T2 relaxometry mapping images in both the Stroke and XSECC treatment group (Fig. 2). T2 values of the ipsilateral cortex and striatum were significantly increased in the Stroke group at all time points, compared with the sham controls. While XSECC obviously down-regulated the increased T2 values after ischemia in the cortex and striatum, suggesting that XSECC has a potential effect on relieving cerebral edema induced by ischemia.

### XSECC ameliorated nerve fiber injuries

**Fig. 3:**
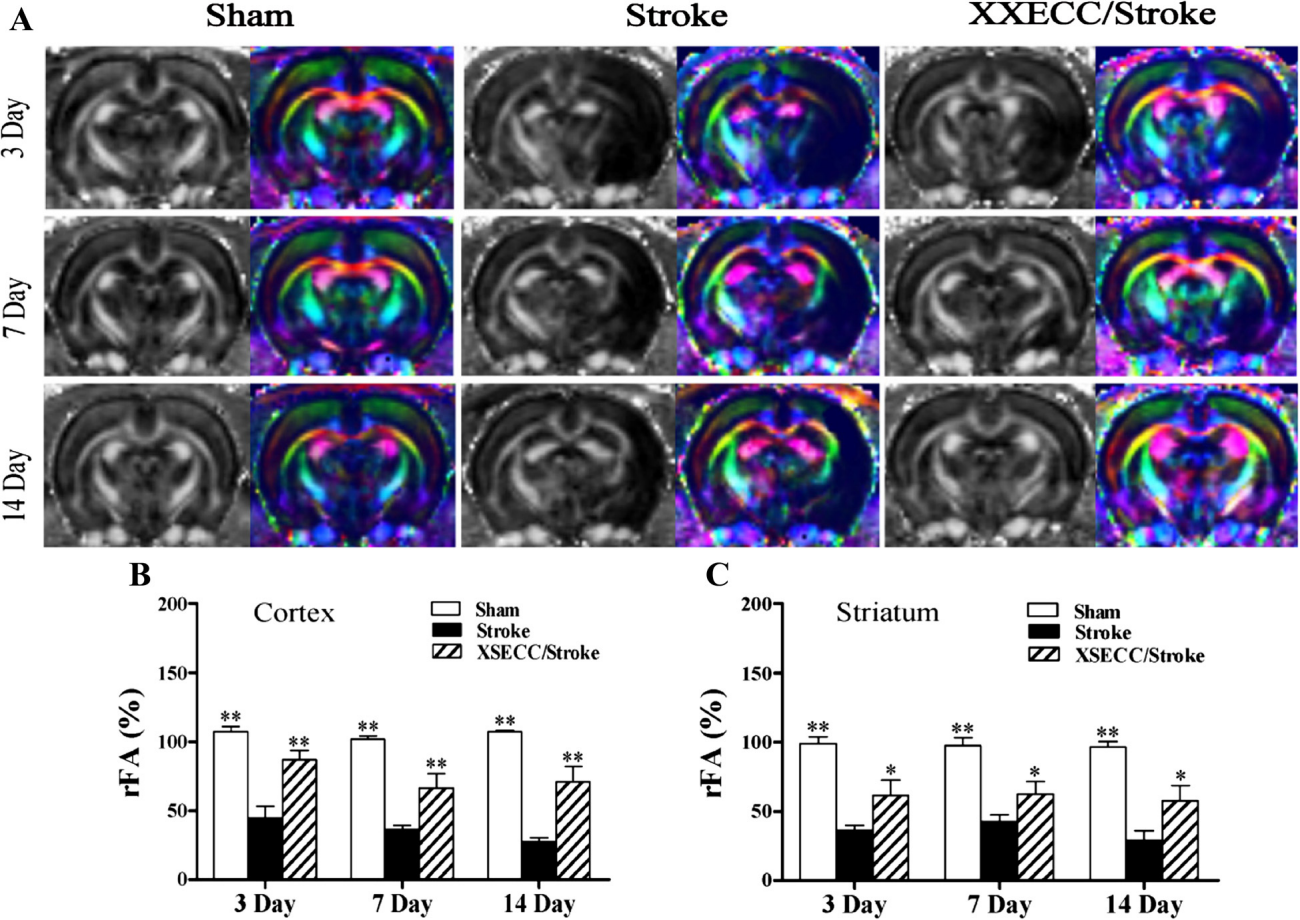
FA images on the 3rd, 7th and 14th days after pMCAO. **a** Representative FA value map and color-encoded FA directional map of the Sham, the Stroke and the XSECC/Stroke groups. rFA in the (**b**) cortex and (**c**) striatum. Values are means ± SEM. *P < 0.05, **P < 0.01 vs the Stroke group

The white matter fiber integrity defined on FA in DTI showed abnormal low signals in the cortex and striatum of all pMCAO rats (Fig. 3). The Stroke group showed decreased rFA on the 3rd, 7th and 14th days in the cortex and striatum, compared with sham controls. XSECC could markedly elevate the rFA in the cortex and striatum, when compared with the Stroke group on the 3rd, 7th, 14th days after ischemia. These data indicate that XSECC could preserve the nerve fiber integrity or delay axonal injury.

### XSECC improved vascular signal strength

**Fig. 4:**
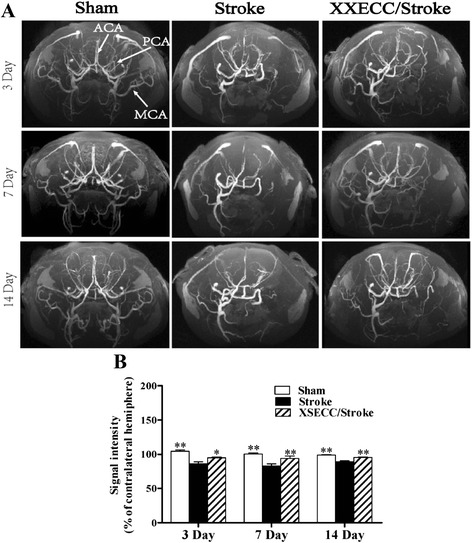
MRA images on the 3rd, 7th and 14th days after pMCAO. **a** Representative MRA images of the Sham, the Stroke and the XSECC/Stroke groups. Anterior cerebral artery (ACA), posterior cerebral artery (PCA), middle cerebral artery (MCA) as indicated by white arrows in the Sham group. **b** Signal intensity of contralateral hemisphere on the 3rd, 7th and 14th days after pMCAO. Values are means ± SEM. *P < 0.05, **P < 0.01 vs the Stroke group

MRA images performed the morphological alterations of arteries on the 3rd, 7th and 14th days (Fig. 4a). The arteries in the Sham group showed normal morphology. The occlusion of MCA in ipsilateral hemisphere was observed in all pMCAO rats. Vascular signal intensity in ipsilateral hemisphere were markedly decreased on the 3rd, 7th and 14th days in the Stroke group, compared with Sham group. XSECC significantly increased signal intensity in ipsilateral hemisphere as compared with the Stroke group on the 3rd, 7th and 14th days (Fig. 4b). These evidences support that XSECC therapy would benefit the recovery of the hemodynamic changes following ischemia.

### XSECC increased the cerebral blood flow

**Fig. 5:**
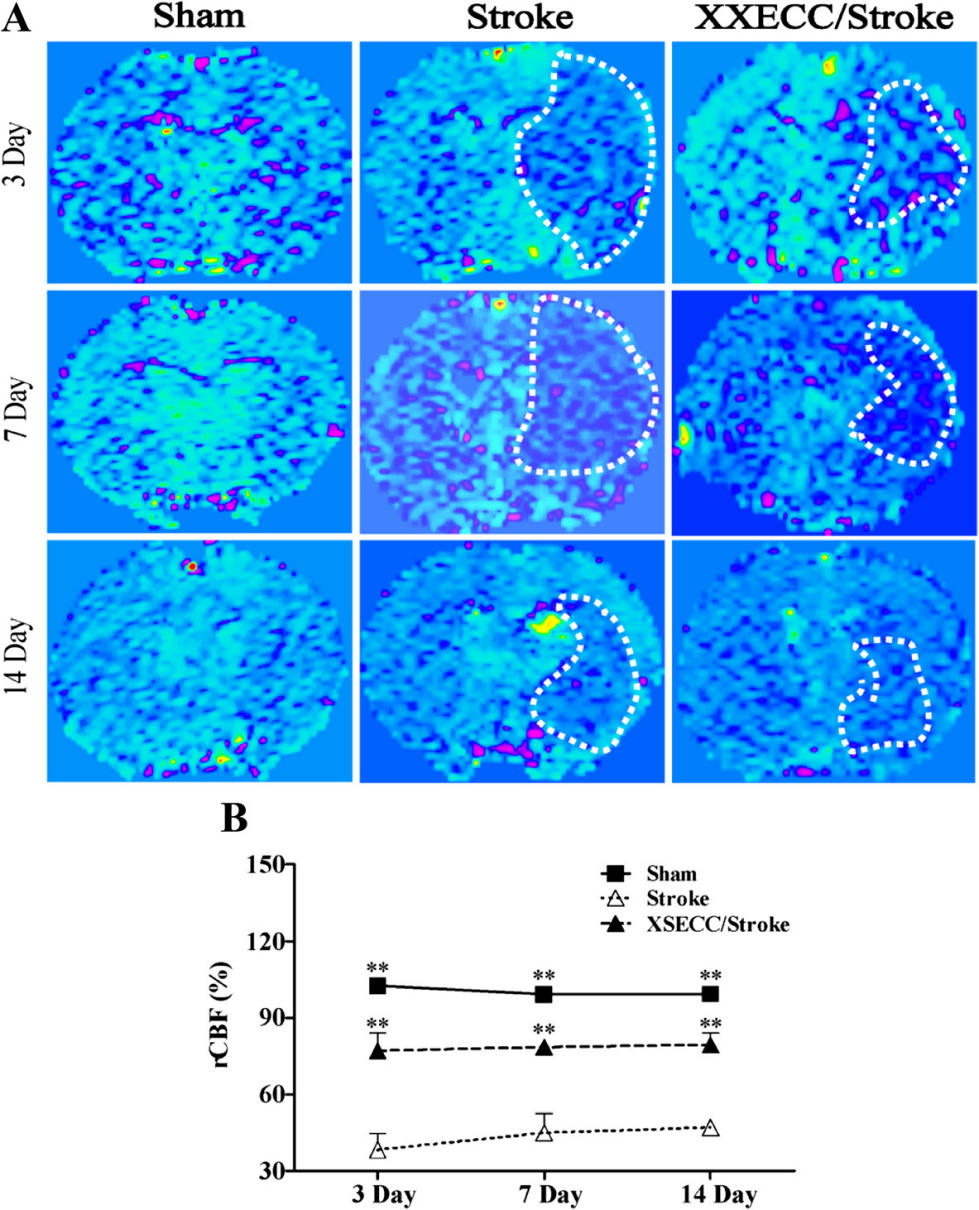
CBF images on the 3rd, 7th and 14th days after pMCAO. **a** Representative CBF images of the Sham, the Stroke and the XSECC/Stroke groups. The low cerebral flow blood as indicated by the white dotted line. **b** rCBF on the 3rd, 7th and 14th days after pMCAO. Values are means ± SEM. **P < 0.01vs the Stroke group

In the ipsilateral hemisphere, CBF images showed a decrease of CBF in the Stroke group. The reduction of CBF was ameliorated by XSECC on the 3rd, 7th and 14th days (Fig. 5a). The statistical analysis showed that rCBF was below 50 % on the 3rd, 7th and 14th days in the Stroke group and there was a significant difference when compared to the Sham group. XSECC treatment increased rCBF to more than 70 % in the ischemic brain region on the 3rd, 7th and 14th days (Fig. 5b), suggesting XSECC could improve hypoperfusion after pMCAO.

### XSECC regulated neuronal/glial metabolism

**Fig. 6:**
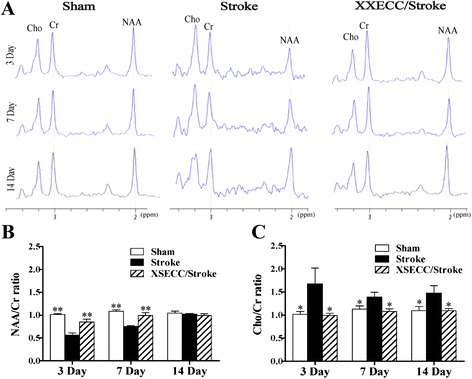
^1^H-MRS on the 3rd, 7th and 14th days after pMCAO. **a** Representative ^1^H-MRS spectra of the Sham, the Stroke and the XSECC/Stroke groups. Cr peak at 3.02 ppm, as an internal reference; Cho peak at 3.22 ppm; NAA peak at 2.02 ppm; (**b**) NAA/Cr ratio and (**c**) Cho/Cr ratio in the ipsilateral hemisphere on the 3rd, 7th and 14th days. Values are means ± SEM. *P < 0.05, **P < 0.01 vs the Stroke group

The relative levels of NAA and Cho peak area in the ipsilateral hemisphere were obtained using ^1^H-MRS assay (Fig. 6a). NAA can be used as a specific biochemical marker to assess neuronal viability/integrity. The level of Cho in the brain tissue indicates the information about glial cell metabolism [35, 36]. In the Stroke group, the NAA/Cr ratio was significantly reduced in the subacute phase (3 – 7 days) after stroke, followed by spontaneous recovery to the normal level on the 14th day after ischemia, while Cho/Cr ratios were obviously increased within 14 days. In XSECC treatment group, the NAA/Cr ratios on the 3rd and 7th days were statistically higher than those in the Stroke group (Fig. 6b). Meanwhile, the Cho/Cr ratios in the XSECC/Stroke group showed statistically lowered at all time points after ischemia, compared with the Stroke group (Fig. 6c). These results suggested that regulating neuronal/glial metabolism might be one of the mechanisms underlying the neurovascular protective effects of XSECC against ischemic injury.

### XSECC protected ultrastructure of NVU

**Fig. 7:**
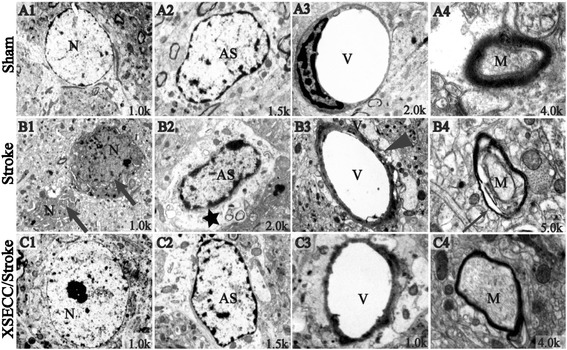
Ultrastructure of the NVU in parietal cortex on the 14th day after pMCAO. Representative images of ultrastructure in (A1-A4) the Sham group, (B1-B4) the Stroke group and (C1-C4) the XSECC/Stroke group. Normal appearance of (A1) neurons and (A2) astrocytes; (A3) the regular capillary; (A4) integral myelin. (B1) Degenerative and necrotic neurons as indicated by broad arrows; (B2) Swollen astrocyte foot processes as indicated by black star; (B3) edema around the capillaries as indicated by black arrow heads and (B4) disrupted Myelin sheath as indicated by narrow arrow. *n* = 2 for each group. N: neurons; AS: astrocytes; V: vascular. M: myelin sheath

The ultrastructural changes of NVU were observed by TEM after pMCAO visually. Neuron, astrocyte, capillary and myelin sheath in the Sham group showed normal structure (Fig. 7). While the Stroke group displayed obviously pathological changes, including neuronal necrosis, swelling of the astrocytes and capillaries as well as damaged myelin sheath which characterized by structural de-arrangement and local demyelination. However, these damages were markedly alleviated in the XSECC/Stroke group, indicating by nerve cell damage, edema around the capillary, swelling of astrocyte foot processes, and myelin sheath degeneration were evidently attenuated after XSECC treatment.

### XSECC up-regulated the expression of NeuN and CD34, but down-regulated GFAP

**Fig. 8:**
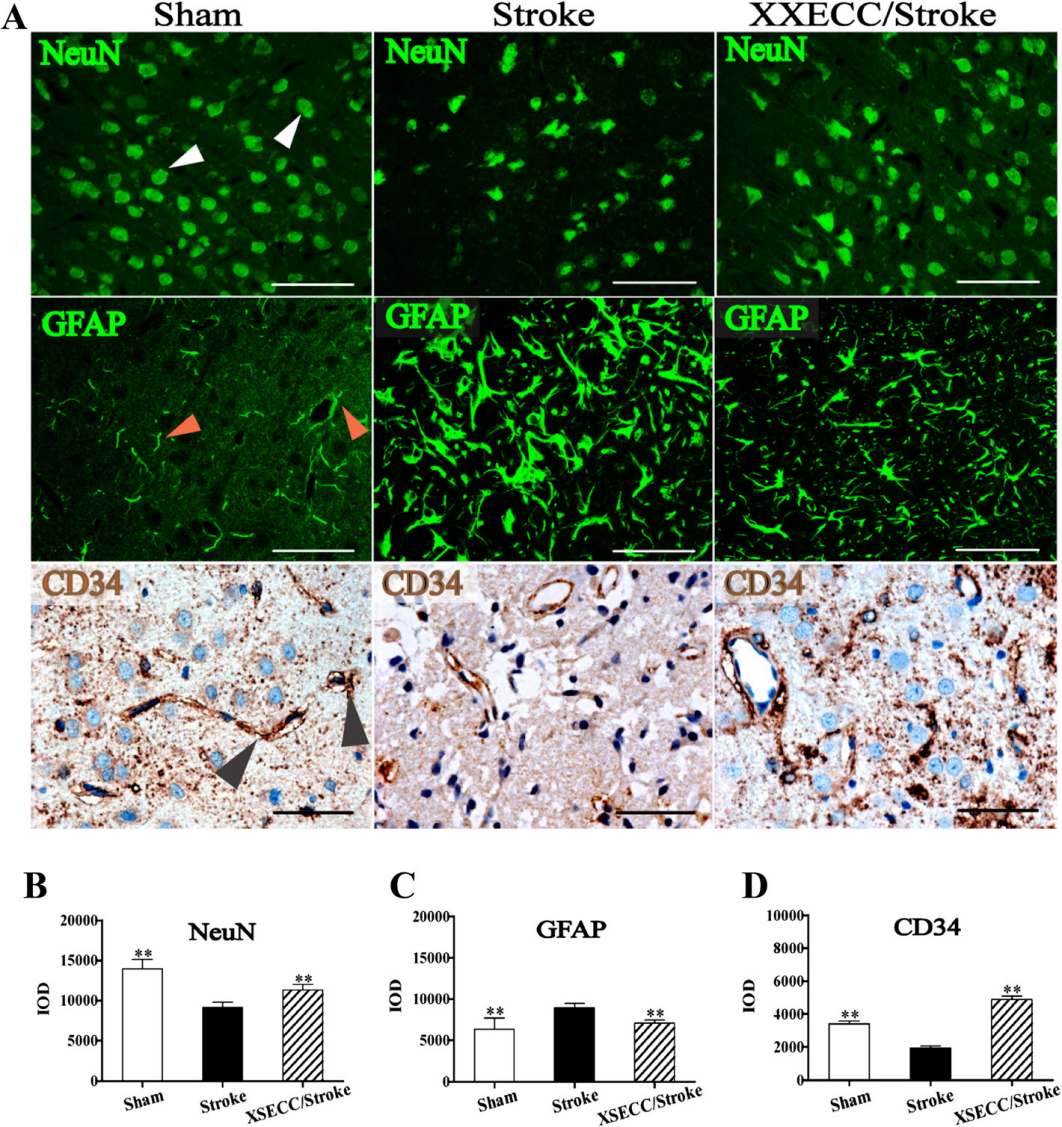
NeuN and GFAP immunofluorescence and CD34 immunohistochemical staining on the 14th day after pMACO. **a** Representative images of NeuN positive neurons, GFAP positive astrocytes and CD34 positive endothelial cells of the Sham, the Stroke and the XSECC/Stroke groups (positive expression of NeuN, GFAP and CD34 as indicated by white, red and black arrow heads, respectively). The expression of (**b**) NeuN, (**c**) GFAP and (**d**) CD34 in the ipsilateral cortex on the14^th^ day after pMACO. Values are means ± SEM (*n* = 4 in the Sham and XSECC/Stroke group; *n* = 3 in the Stroke group). **P < 0.01 vs the Stroke group. Scale bar = 50 μm

Figure 8 showed that the expressions of NeuN-positive neurons, CD34-positive endothelial cells and GFAP-positive astrocytes were down-regulated in the peri-infarct cortex of the Stroke group on the 14th day after ischemia. However, XSECC treatment up-regulated NeuN protein expression and generated CD34-positive capillaries in the peri-infarct region on the 14th day after stroke (Fig. 8b and d), indicating that XSECC not only ameliorated the neuron injury, but also reduced endothelial damage. Additionally, the expression levels of GFAP in the ischemic cortex were significantly down-regulated in XSECC-treated rats (Fig. 8c), which suggested that XSECC might inhibit the astrocyte proliferation (astrogliosis). The findings provided strong evidence that XSECC had multi-target NVU protective effects aganist ischemic brain injury.

## Discussion

Neurovascular dysfunction after stroke is multifaceted, which contributes to a broad pathological and functional impairment in the ischemic brain [23]. Using multiparametric MRI, we could visualize the development of neurovascular pathology following pMCAO. This research has improved our understanding of the neurovascular protective effects of XSECC against ischemic brain damage.

In this study, multiparametric MRI (T2WI, T2 relaxometry mapping and DTI) was used to monitor the impairment of neurovascular functions. We found abnormally high signals in T2WI images in the ipsilateral hemisphere of pMCAO rats between the 3rd and 14th days. Hyperintensity on T2 relaxometry mapping images in the same area was also observed at the same time points. XSECC could reduce the cerebral infarct volume between the 3rd and 14th days post-ischemia. Additionally, T2 values within injured cortical and striatal regions were significantly decreased, indicating that XSECC has an obvious effect in relieving edema after an ischemic insult. It is well known that a severe FA decline can be associated with a loss of membrane integrity, axonal degeneration and demyelination during pMCAO [31, 42–44]. In our study, abnormal FA hypointense signals were observed in the right striatum and cortex of the pMCAO rats. Compared to the stroke group, there was an obvious increase in the FA signal intensity in XSECC group on the 3rd, 7th and 14th days, suggesting that application of XSECC after stroke might preserve the integrity of nerve fibers and reduce axonal degeneration.

NVU consists of micro-vessels, astrocytes, neurons, axons, and other supporting cells [4]. To investigate the microscopic structural changes of NVU, we determined the expression changes of NVU markers, including NeuN (neuronal marker), GFAP (glial cell marker), and CD34 (vascular endothelial cell maker) on the 14th day after cerebral ischemia. A rapid loss of NeuN, increased GFAP expression as well as decreased vascular endothelial cells were observed after pMCAO. Combined with the changes of NVU ultrastructural investigation by TEM, we found that neurovascular dysfunction evolved with angioedema, axonal loss, myelin vacuolization and demyelinization. Interestingly, treatment with XSECC enhanced neuronal and endothelial cell survival and suppressed astrocytic proliferation (astrogliosis), suggestive of remarkable neurovascular protective effects.

In short, T2WI, T2 relaxometry mapping and FA quantitative assessment provide valuable non-invasive information about NVU functions injury, which is consistent with histopathological and ultrastructural changes of the NVU after stroke. These MRI‑based findings could offer a relatively simple observation to confirm the NVU protective effects of XSECC against ischemic injury.

Next, we would further investigate how XSECC exerted its NVU protection against cerebral ischemia. It is well accepted that NVU plays an important role in supporting the cerebral functions by regulating its perfusion and metabolism through the combined efforts of neurons, astrocytes, and vascular endothelial cells [23]. Hence, a further aim of this study was to evaluate the effects of XSECC on neurochemical metabolite levels and vascular perfusion following pMCAO.

^1^H-MRS can supply the information about neuronal metabolites changes, including neuronal health, gliosis, osmoregulation, energy metabolism, and neuronal-glial cycling [35]. In our present study, ^1^H-MRS was designed to monitor the changes of metabolites after XSECC treatment. NAA, a commonly used marker for neuronal integrity and healthy neuronal function, shows a dynamic changes after stroke [36]. Reductions of NAA may suggest neuronal degeneration or loss [36]. In addition to neuronal impairment, efflux of intracellular NAA in response to ischemia-induced cytotoxic edema and subsequent clearance into the blood circulation might also contributes to the initial NAA loss [45]. Moreover, Cho is a mixture of molecules containing tri-methylamine being predominantly present in cell membranes, which reflects the degree of myelinization, proliferation and membrane functions [35]. Elevated Cho levels suggested myelin dissolution and astrocytic gliosis [35, 46]. In our study, NAA/Cr ratios were obviously decreased on the 3rd and 7th days, but the Cho/Cr ratios in the lesion areas were increased over time after pMCAO, which indicates neuronal injury and the activation of astrocytes. However, XSECC remarkably elevated NAA/Cr ratios on the 3rd and 7th days, but lowered Cho/Cr ratios at all time points in the ipsilateral hemisphere after cerebral ischemia. These results are consistent with the histological findings, which implied that XSECC could protect neurons, inhibit gliosis and maintain nerve cell membrane function. Therefore, the metabolic results suggested that the protective effects of XSECC against NVU damage may be, at least in part, attributed to the regulation of neuronal/glial metabolism.

Since the neurovascular abnormalities might be related to altered vascular perfusion [23, 26], we studied the temporal evolution of neurovascular functions by 3D TOF-MRA images in combination with quantitative ASL-CBF measurements to determine the effects of XSECC on blood perfusion state in ischemic region. The 3D TOF-MRA is one of the most advanced imaging techniques used to monitor the morphologic changes of intracranial arteries in vivo [34]. Furthermore, ASL perfusion MR imaging is a noninvasive method for measuring CBF directly by using labeled arterial blood as an endogenous tracer [47]. Our results showed that decreased MRA signal intensity and CBF in the MCA blood supply area, was related to arterial vessel occlusion after stroke, while significant increase in MRA signal intensity and CBF were observed on the 14th day after XSECC treatment. Coincident with these MRI changes, histological inspection showed XSECC obviously up-regulated CD34 expression and lightened vascular edema on the 14th day after pMACO. These results indicated that XSECC might play a role in neurovascular protection by promoting vascular remodeling and improving CBF after pMCAO.

To date, tissue plasminogen activator (tPA) is the only thrombolytic treatment for acute ischemic stroke approved by the U.S. FDA. tPA administered within 4.5 h of symptom onset dissolves the fibrin contained in a clot to re-establish blood flow [23]. However, only a small percentage of stroke patients are eligible for tPA, which largely due to the narrow therapeutic window and dangerous side effects [48]. Some studies suggested that tPA promotes neurovascular dysfunction by facilitating blood brain barrier disruption [49], stimulating excitotoxic neurons [50], increasing oxidative stress and inducing inflammatory responses [51]. Therefore, pharmacological agent like XSECC, which potentiates neurovascular function may enhance the efficacy and safety of thrombolytic therapy [23].

Based on the traditional documentation of BYHWD, XSECC is prepared from seven medical herbs, including Radix Astragali, Radix Angelicae Sinensis, Radix Paeoniae Rubra, Rhizoma Ligustici Chuanxiong, Flos Carthami, Semen Persicae and Pheretima. All the herbs are recorded in the Chinese Pharmacopoeia. Studies have found that some of the active constituents isolated from these crude herbs have potent vascular protective and anti-thrombotic effects, including improving endothelial function, inhibiting platelet activation and antagonizing coagulation cascades [13]. For example, Lumbrokinase extracted from Pheretima, is a strong fibrinolytic enzyme and can be absorbed from mucosal lumen into blood by oral administration [52]. Astragaloside IV, which used as a marker compound for quality control of Radix Astragali, can increase fibrinolytic potential not only by up-regulating the expression of tPAr, but also by dampening the expression of plasminogen activator inhibitor-1 [53]. Paeoniflorin is one of the principal compound of Radix Paeoniae Rubra, has the capability to inhibit VSMC proliferation and the expression of platelet-derived growth factor in vivo [54]. Tetramethylpyrazine is a marking composition of Rhizoma Ligustici Chuanxiong, has the wide range of pharmacological actions such as inhibiting phosphoinositide breakdown and thromboxane A2 formation, as well as attenuating platelet aggregation [54, 55]. These studies suggested that XSECC exhibits neurovascular effects through multiple components aimed at multiple targets and mechanisms.

In summary, we confirmed that XSECC has a potent neurovascular protection when administration after ischemic stroke. XSECC could restore the normal function of NVU by regulating neuronal/glial metabolism and re-establishing CBF. The results provide a basis for further study on the neurovascular protective effects of XSECC.

## Abbreviations

^1^H-MRS, ^1^H-magnetic resonance spectroscopy; ASL, arterial spin labeling; BYHWD, *Buyang Huanwu* Decoction; CBF, cerebral blood flow; Cho, choline; Cr, crestine; DTI, diffusion tensor imaging; FA, fractional anisotropy; GFAP, glial fibrillary acidic protein; IOD, integrated optical density; MRA, magnetic resonance angiography; MRI, magnetic resonance imaging; NAA, N-acetylaspartate; NeuN, neuron specific nuclear protein; NVU, neurovascular unit; pMCAO, permanent middle cerebral artery occlusion; SEM, standard error of the mean; T2WI, T2 weighted imaging; TCM, Traditional Chinese Medicine; TEM, transmission electron microscopy; XSECC, *Xiaoshuan* enteric-coated capsule

## References

[1] Faralli A, Bigoni M, Mauro A, Rossi F, Carulli D. Noninvasive strategies to promote functional recovery after stroke. Neural Plast. 2013; doi:10.1155/2013/854597. 10.1155/2013/854597 PMC3707231 23864962

[2] Pandya RS, Mao L, Zhou H, Zhou S, Zeng J, Popp AJ (2011). Central nervous system agents for ischemic stroke: neuroprotection mechanisms. Cent Nerv Syst Agents Med Chem.

[3] Nathaniel TI, Williams-Hernandez A, Hunter AL, Liddy C, Peffley DM, Umesiri FE (2015). Tissue hypoxia during ischemic stroke: adaptive clues from hypoxia-tolerant animal models. Brain Res Bull.

[4] Zhou Z, Wei X, Xiang J, Gao J, Wang L, You J (2015). Protection of erythropoietin against ischemic neurovascular unit injuries through the effects of connexin43. Biochem Biophys Res Commun.

[5] Pan J, Lei X, Wang J, Huang S, Wang Y, Zhang Y (2015). Effects of Kaixinjieyu, a Chinese herbal medicine preparation, on neurovascular unit dysfunction in rats with vascular depression. BMC Complement Altern Med.

[6] Lan R, Xiang J, Zhang Y, Wang GH, Bao J, Li WW, et al. PI3K/Akt Pathway Contributes to Neurovascular Unit Protection of Xiao-Xu-Ming Decoction against Focal Cerebral Ischemia and Reperfusion Injury in Rats. Evid Based Complement Alternat Med. 2013; doi: 10.1155/2013/459467 10.1155/2013/459467 PMC3678438 23781261

[7] del Zoppo GJ (2010). The neurovascular unit in the setting of stroke. J Intern Med.

[8] Vangilder RL, Rosen CL, Barr TL, Huber JD (2011). Targeting the neurovascular unit for treatment of neurological disorders. Pharmacol Ther.

[9] Ginsberg MD (2009). Current status of neuroprotection for cerebral ischemia: synoptic overview. Stroke.

[10] Lo EH (2008). A new penumbra: transitioning from injury into repair after stroke. Nat Med.

[11] Shen J, Zhu Y, Yu H, Fan ZX, Xiao F, Wu P (2014). Buyang Huanwu decoction increases angiopoietin-1 expression and promotes angiogenesis and functional outcome after focal cerebral ischemia. J Zhejiang Univ Sci.

[12] Kong X, Su X, Zhu J, Wang J, Wan H, Zhong M (2014). Neuroprotective effect of buyang huanwu decoction on rat ischemic/reperfusion brain damage by promoting migration of neural precursor cells. Rejuvenation Res.

[13] Li JH, Liu AJ, Li HQ, Wang Y, Shang HC, Zheng GQ (2014). Buyang huanwu decoction for healthcare: evidence-based theoretical interpretations of treating different diseases with the same method and target of vascularity. Evid Based Complement Altern Med.

[14] Qu TB, Yu TH, Liu ZT, Li L, Chu LS (2014). Effect of Buyang Huanwu Decoction and its disassembled recipes on rats’ neurogenesis after focal cerebral ischemia. Zhongguo Zhong xi yi jie he za zhi.

[15] Sun J, Bi Y, Guo L, Qi X, Zhang J, Li G (2007). Buyang Huanwu Decoction promotes growth and differentiation of neural progenitor cells: using a serum pharmacological method. J Ethnopharmacol.

[16] Wang HW, Liou KT, Wang YH, Lu CK, Lin YL, Lee IJ (2011). Deciphering the neuroprotective mechanisms of Bu-yang Huan-wu decoction by an integrative neurofunctional and genomic approach in ischemic stroke mice. J Ethnopharmacol.

[17] Zhao LD, Wang JH, Jin GR, Zhao Y, Zhang HJ (2012). Neuroprotective effect of Buyang Huanwu decoction against focal cerebral ischemia/reperfusion injury in rats--time window and mechanism. J Ethnopharmacol.

[18] Jinglong T, Weijuan G, Jun L, Tao Q, Hongbo Z, Shasha L (2013). The molecular and electrophysiological mechanism of buyanghuanwu decoction in learning and memory ability of vascular dementia rats. Brain Res Bull.

[19] Cai G, Liu B, Liu W, Tan X, Rong J, Chen X (2007). Buyang Huanwu Decoction can improve recovery of neurological function, reduce infarction volume, stimulate neural proliferation and modulate VEGF and Flk1 expressions in transient focal cerebral ischaemic rat brains. J Ethnopharmacol.

[20] Wu L, Zhang W, Li H, Chen BY, Zhang GM, Tang YH (2009). Inhibition of aortic intimal hyperplasia and cell cycle protein and extracellular matrix protein expressions by BuYang HuanWu Decoction. J Ethnopharmacol.

[21] Wang YL, Zhang N, Liu J, Wu X, Wang L, Zhao H (2014). Effects of Xiaoshuan Enteric-coated Capsule on Astrocyte Activation and Expression of Caspase-3, PARP in Remote Encephalic Region of MCAO Rats. Chin J Stroke.

[22] Zhao H, Wang L, Wang CX, Zhang N, Zhang J, Wang YL (2014). The effects of Xiaoshuan enteric-coated capsule on novelty seeking behavior and dendrite reconstruction in middle cerebral artery occlusion rat. Chin J Neuroimmunol Neurol.

[23] Zhang L, Zhang ZG, Chopp M (2012). The neurovascular unit and combination treatment strategies for stroke. Trends Pharmacol Sci.

[24] Nagel S, Wagner S, Koziol J, Kluge B, Heiland S (2004). Volumetric evaluation of the ischemic lesion size with serial MRI in a transient MCAO model of the rat: comparison of DWI and T1WI. Brain Res Brain Res Protoc.

[25] Lee JH, Lee YK, Ishikawa M, Koga K, Fukunaga M, Miyakoda G (2003). Cilostazol reduces brain lesion induced by focal cerebral ischemia in rats--an MRI study. Brain Res.

[26] Chen CC, Chen YC, Hsiao HY, Chang C, Chern Y (2013). Neurovascular abnormalities in brain disorders: highlights with angiogenesis and magnetic resonance imaging studies. J Biomed Sci.

[27] Longa EZ, Weinstein PR, Carlson S, Cummins R (1989). Reversible middle cerebral artery occlusion without craniectomy in rats. Stroke.

[28] Huang YC, Tzeng WS, Wang CC, Cheng BC, Chang YK, Chen HH (2013). Neuroprotective effect of agmatine in rats with transient cerebral ischemia using MR imaging and histopathologic evaluation. Magn Reson Imaging.

[29] Badaut J, Ashwal S, Tone B, Regli L, Tian HR, Obenaus A (2007). Temporal and regional evolution of aquaporin-4 expression and magnetic resonance imaging in a rat pup model of neonatal stroke. Pediatr Res.

[30] He Z, Wang X, Wu Y, Jia J, Hu Y, Yang X (2014). Treadmill pre-training ameliorates brain edema in ischemic stroke via down-regulation of aquaporin-4: an MRI study in rats. PLoS One.

[31] Tuor UI, Morgunov M, Sule M, Qiao M, Clark D, Rushforth D (2014). Cellular correlates of longitudinal diffusion tensor imaging of axonal degeneration following hypoxic-ischemic cerebral infarction in neonatal rats. Neuroimage Clin.

[32] Guo J, Zheng HB, Duan JC, He L, Chen N, Gong QY (2011). Diffusion tensor MRI for the assessment of cerebral ischemia/reperfusion injury in the penumbra of non-human primate stroke model. Neurol Res.

[33] Iskander A, Knight RA, Zhang ZG, Ewing JR, Shankar A, Varma NR (2013). Intravenous administration of human umbilical cord blood-derived AC133+ endothelial progenitor cells in rat stroke model reduces infarct volume: magnetic resonance imaging and histological findings. Stem Cells Transl Med.

[34] Besselmann M, Liu M, Diedenhofen M, Franke C, Hoehn M (2001). MR angiographic investigation of transient focal cerebral ischemia in rat. NMR Biomed.

[35] Choi JK, Dedeoglu A, Jenkins BG (2007). Application of MRS to mouse models of neurodegenerative illness. NMR Biomed.

[36] Igarashi H, Suzuki Y, Huber VJ, Ida M, Nakada T (2015). N-acetylaspartate decrease in acute stage of ischemic stroke: a perspective from experimental and clinical studies. Magn Reson Med Sci.

[37] Monkul ES, Yildiz A, Soares C (2004). Magnetic resonance spectroscopy (MRS) applications in bipolar disorder. Turk Psikiyatri Derg.

[38] Wang H, Wang L, Zhang N, Zhang Q, Zhao H (2014). Houshiheisan compound prescription protects neurovascular units after cerebral ischemia. Neural Regen Res.

[39] Lan R, Zhang Y, Xiang J, Zhang W, Wang GH, Li WW (2014). Xiao-Xu-Ming decoction preserves mitochondrial integrity and reduces apoptosis after focal cerebral ischemia and reperfusion via the mitochondrial p53 pathway. J Ethnopharmacol.

[40] Ye YL, Shi WZ, Zhang WP, Wang ML, Zhou Y, Fang SH (2007). Cilostazol, a phosphodiesterase 3 inhibitor, protects mice against acute and late ischemic brain injuries. Eur J Pharmacol.

[41] Ortega FJ, Jolkkonen J, Mahy N, Rodriguez MJ (2013). Glibenclamide enhances neurogenesis and improves long-term functional recovery after transient focal cerebral ischemia. J Cereb Blood Flow Metab.

[42] Cengiz P, Uluc K, Kendigelen P, Akture E, Hutchinson E, Song C (2011). Chronic neurological deficits in mice after perinatal hypoxia and ischemia correlate with hemispheric tissue loss and white matter injury detected by MRI. Dev Neurosci.

[43] Kim JH, Na DG, Chang KH, Song IC, Choi SH, Son KR (2013). Serial MR analysis of early permanent and transient ischemia in rats: diffusion tensor imaging and high b value diffusion weighted imaging. Korean J Radiol.

[44] Liu Y, D’Arceuil HE, Westmoreland S, He J, Duggan M, Gonzalez RG (2007). Serial diffusion tensor MRI after transient and permanent cerebral ischemia in nonhuman primates. Stroke.

[45] Taylor DL, Davies SE, Obrenovitch TP, Doheny MH, Patsalos PN, Clark JB (1995). Investigation into the role of N-acetylaspartate in cerebral osmoregulation. J Neurochem.

[46] Oguzhanoglu NK, Sozeri-Varma G, Karadag F, Tumkaya S, Efe M, Kiroglu Y (2014). Prefrontal cortex neurochemical metabolite levels in major depression and the effects of treatment: an HMRS study. Turk Psikiyatri Derg.

[47] Niibo T, Ohta H, Yonenaga K, Ikushima I, Miyata S, Takeshima H (2013). Arterial spin-labeled perfusion imaging to predict mismatch in acute ischemic stroke. Stroke.

[48] Adeoye O, Hornung R, Khatri P, Kleindorfer D (2011). Recombinant tissue-type plasminogen activator use for ischemic stroke in the United States: a doubling of treatment rates over the course of 5 years. Stroke.

[49] Montaner J, Molina CA, Monasterio J, Abilleira S, Arenillas JF, Ribo M (2003). Matrix metalloproteinase-9 pretreatment level predicts intracranial hemorrhagic complications after thrombolysis in human stroke. Circulation.

[50] Lo EH, Broderick JP, Moskowitz MA (2004). tPA and proteolysis in the neurovascular unit. Stroke.

[51] Warach S, Latour LL (2004). Evidence of reperfusion injury, exacerbated by thrombolytic therapy, in human focal brain ischemia using a novel imaging marker of early blood–brain barrier disruption. Stroke.

[52] Yan XM, Kim CH, Lee CK, Shin JS, Cho IH, Sohn UD (2010). Intestinal absorption of fibrinolytic and proteolytic lumbrokinase extracted from Earthworm. Eisenia Andrei Korean J Physiol Pharmacol.

[53] Zhang WJ, Wojta J, Binder BR (1997). Regulation of the fibrinolytic potential of cultured human umbilical vein endothelial cells: astragaloside IV downregulates plasminogen activator inhibitor-1 and upregulates tissue-type plasminogen activator expression. J Vasc Res.

[54] Ji B, Geng P, Liu JG, Shi DZ, Wang YY (2006). Effects of active components extracted from Qixue Bingzhi Recipe on proliferation of vascular smooth muscle cells and expressions of platelet-derived growth factor and its receptor genes. Zhong Xi Yi Jie He Xue Bao.

[55] Sheu JR, Kan YC, Hung WC, Ko WC, Yen MH (1997). Mechanisms involved in the antiplatelet activity of tetramethylpyrazine in human platelets. Thromb Res.

